# Associations between maternal fish intake and polyunsaturated fatty acid status with childhood asthma in a high fish‐eating population

**DOI:** 10.1111/pai.70019

**Published:** 2025-01-08

**Authors:** Cealan O. Henry, Philip J. Allsopp, Alison J. Yeates, Toni Spence, Marie C. Conway, Maria S. Mulhern, Emelyn Shroff, Conrad F. Shamlaye, Juliette Henderson, Edwin van Wijngaarden, Gary J. Myers, J. J. Strain, Emeir M. McSorley

**Affiliations:** ^1^ The Nutrition Innovation Centre for Food and Health, School of Biomedical Sciences Ulster University Coleraine Northern Ireland UK; ^2^ Ministry of Health Mahé Island Republic of Seychelles; ^3^ The School of Medicine and Dentistry University of Rochester Rochester New York USA

**Keywords:** asthma, cord, docosahexaenoic acid, fish, maternal, polyunsaturated fatty acids

## Abstract

**Background:**

Studies investigating associations between prenatal polyunsaturated fatty acid status (PUFAs), in particular the anti‐inflammatory n‐3 PUFAs, and the development of childhood asthma have yielded conflicting results.

**Objective:**

To determine the associations between maternal fish intake (a rich source of the n‐3 PUFAs), maternal or cord PUFAs with the prevalence of childhood asthma in a high fish‐eating population.

**Methods:**

We examined these associations between fish intake and PUFA concentrations with childhood asthma prevalence in the Seychelles Child Development Study Nutrition Cohort 2, a large observational study in a high fish‐eating population. Maternal fish intake during pregnancy and child's fish intake at 7 years were assessed by questionnaire, with frequency reported as meals/week. Serum concentrations of PUFAs were quantified in maternal blood collected at 28 weeks' gestation (*n* = 1448) and in cord blood (*n* = 1088). Asthma in children at 7 years was assessed using the International Study of Asthma and Allergies in Childhood (ISAAC) questionnaire (*n* = 1098).

**Results:**

A total of 97 children (10.5%) were reported to have asthma. In regression analysis, the odds of childhood asthma were not associated with maternal fish intake or maternal PUFA status. Cord DHA concentrations were associated with increased asthma prevalence when the highest quartile (≥0.123 mg/mL) was compared with the lowest (<0.061 mg/mL).

**Conclusion:**

The results from this current study add to the growing body of evidence that fish consumption during pregnancy is not associated with asthma development in offspring. The associations between cord blood DHA and asthma prevalence are unexpected and warrant further investigation.

**Abbreviations**
ALA: Alpha‐linolenic acid
AA: arachidonic acid
DHA: docosahexaenoic acid
EPA: eicosapentaenoic acid
SES: Hollingshead socioeconomic status
ISAAC: International Study of Asthma and Allergies in Childhood
PUFAs: polyunsaturated fatty acids
SCDS NC2: Seychelles Child Development Study Nutrition Cohort 2

**Key message**

High consumption of fish during pregnancy is not associated with asthma development in childhood, adding to the literature gap surrounding maternal fish consumption and childhood asthma. The association identified between higher cord blood DHA and increased asthma prevalence is unexpected and requires further exploration.

## 1 INTRODUCTION

Asthma is a common chronic inflammatory airway disease affecting over 300 million people worldwide.^1^, ^2^ Increased asthma prevalence has been reported in children from westernized populations,^3^ with higher rates identified in male children.^4^ Evidence suggests that in utero environmental exposures may be implicated in the development of asthma in later life.^5^ In particular, the maternal diet is believed to play a significant role in the development and maturation of the child's immune system in utero; thereby influencing their asthma susceptibility.^6^

In 1997, Black & Sharpe hypothesized that the higher intake of n‐6 polyunsaturated fatty acids (PUFAs), in comparison to n‐3 PUFAs, might explain the increase in childhood asthma prevalence.^7^ The optimal n6:n3 ratio is approximately 1–2:1 for normal physiological function, although typical western diets have a ratio around 16:1.^8^ Owing to the high abundance of n‐6 PUFAs within western diets, there is a higher rate of n‐6 PUFA conversion displacing n‐3 PUFAs from cell membranes and leading to increased production of pro‐inflammatory prostaglandins and leukotrienes, resulting in allergic immune responses.^6^

Recently, an American cohort of 1019 mother–child dyads observed higher maternal n‐6 PUFA status was associated with increased childhood asthma prevalence, and higher maternal n‐3 PUFAs reduced asthma risk.^9^ Yet, other birth cohort's findings have been inconsistent. Specifically, a birth cohort conducted in the United Kingdom found higher n‐3 PUFAs in maternal plasma phospholipids were associated with reduced asthma prevalence in offspring; no significant observations were identified with maternal n‐6 PUFAs.^10^ In a Dutch cohort, higher maternal n‐6 PUFAs were associated with reduced asthma prevalence in children, while maternal n‐3 PUFA status had no significant impact.^11^ No significant observations were found between maternal n‐6 or n‐3 PUFA status with childhood asthma prevalence in a separate Dutch cohort.^12^

Increasing consumption of n‐3 PUFA dietary sources or supplementation is thought to yield less inflammatory prostaglandins and leukotrienes and increase production of pro‐resolving mediators.^13^ Studies that have investigated the associations between fish consumption (a rich source of n‐3 PUFAs) during pregnancy with childhood asthma prevalence have reported inconsistent findings. Some have demonstrated a protective effect against asthma development,^14^, ^15^, ^16^ while others have reported null associations.^17^, ^18^ Intervention studies with n‐3 PUFA supplementation during pregnancy, however, have reported that an increased in n‐3 PUFA status was associated with lower prevalence of asthma in offspring.^19^, ^20^, ^21^

Evidence highlighted above determined maternal PUFA status in plasma phospholipids. The known mechanism of transport of PUFAs across the placenta is in the form of non‐esterified triacylglycerols quantifiable in serum.^22^ Furthermore, the previous studies only accounted for maternal PUFA status, with a multitude of factors affecting the transfer of PUFA across the placenta. Therefore, determining cord blood PUFA composition may be a better reflection on the relationship between PUFAs and asthma development. Research to date reporting the associations between prenatal exposure to fish with childhood asthma have been performed in cohorts consuming circa 2 fish meals or less per week, and without supplementation this intake would result in low n‐3 PUFA status. Taken altogether, no plausible conclusions can be drawn, warranting further research.

A population of interest to examine the relationship between maternal fish consumption and n‐3 PUFA status with asthma prevalence would be in a cohort of mother–child dyads of frequent fish consumption and subsequently a higher n‐3 PUFA status. The Seychellois population is regarded as high fish consumers (~12 meals/week), with fish being their primary source of protein.^23^ Using data from the Seychelles Child Development Study Nutrition Cohort 2 (SCDS NC2), we examined the associations between maternal fish consumption during pregnancy and children's fish consumption at 7 years of age, maternal and cord blood PUFA status, and the prevalence of asthma in children aged 7 years. We also examined the interactions between PUFA status (maternal or cord) and child sex with asthma prevalence.

## 2 METHODS

### 2.1 Study population

The SCDS NC2 recruited pregnant women at their first prenatal visit (~14 weeks gestation) during 2008–2011.^24^ Recruitment took place in eight centres across Mahé, the largest island in the Republic of Seychelles. Inclusion criteria included being native Seychellois, over 16 years of age, having a singleton pregnancy and having no obvious health concerns. Ethical approval for the study was obtained from the Seychelles Ethics Board and the Research Subject Review Board at the University of Rochester. Written informed consent was obtained from all participants. The study was conducted according to the guidelines laid down in the Declaration of Helsinki.

### 2.2 Fish consumption data

Mothers completed a retrospective fish use questionnaire (FUQ) at 28 weeks' gestation. The questionnaire included fish species native to the Seychellois population, and frequency was reported as meals eaten per week. These data were categorized for the purpose of analysis as oily fish, lean fish, and total fish intake (sum of oily, lean, and other seafood products).^25^ In addition, information regarding the child's fish intake was collected at the 7‐year re‐enrollment visit by having the caregiver(s) complete the FUQ for the child.

### 2.3 PUFA analysis

The PUFA analysis was described in detail previously.^24^ Briefly, maternal blood samples were drawn at the 28‐week visit, and cord blood samples were collected at delivery, from which serum was processed, aliquoted, and stored at −80°C until analysis. Using the gold‐standard technique of gas‐chromatography‐mass spectrometry (7890A‐5975C; Agilent) with the internal standard heptadecanoic acid (C17:0), fatty acid methyl esters were detected and quantified in serum using an adapted method.^26^ The individual PUFAs included in the analysis were arachidonic acid (AA; C20:4 n‐6), linoleic acid (LA; C18:2 n‐6), alpha‐linolenic acid (ALA; C18:3 n‐3), eicosapentaenoic acid (EPA; C20:5 n‐3), and docosahexaenoic acid (DHA; C22:6 n‐3).

### 2.4 Measure of asthma‐related outcomes

**FIGURE 1:**
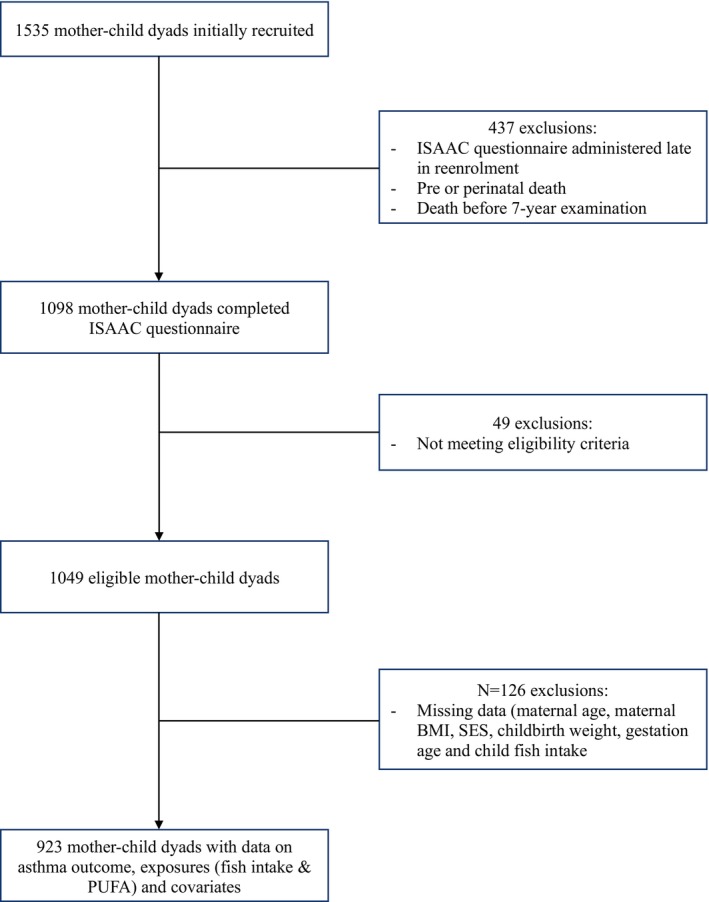
Descriptive of exclusions and missing data for regression analysis. BMI, body mass index; ISAAC, International Study of Asthma and Allergies in Childhood; SES, Hollingshead socioeconomic status.

When the children of the NC2 cohort were about 7 years of age (range, 7.0–7.9 years), the caregiver(s) were asked to complete the validated International Study of Asthma and Allergies in Childhood (ISAAC) questionnaire.^27^ Asthma presence in the child was defined based on an affirmative response to two out of the three following questions: “Has your child had wheezing or whistling in the chest in the past 12 months?”, “Has your child ever had asthma?” and “Has a doctor/medical professional ever prescribed your child medication for asthma?” (adapted^9^). A total of 1098 mother–child dyads completed the ISAAC questionnaire, owing to the questionnaire being administered late into the re‐enrollment of participants and along with other exclusion criteria outlined in Figure 1.

### 2.5 Statistical analysis

Statistical analysis was conducted using SPSS version 29.0 (SPSS Inc., Armonk, NY, USA). Descriptive statistics, including maternal weight, height, BMI (BMI = weight (kg)/height (m)^2^), and child characteristics, are reported using means and standard deviations, unless stated otherwise. The Kolmogorov–Smirnov test was used to assess the distribution of maternal fish intake, maternal PUFA status, and cord blood PUFA concentrations. The maternal and cord PUFA concentrations were not normally distributed, and therefore, differences between asthmatic and non‐asthmatic mother–child dyads were compared using Mann–Whitney *U* and reported as median (25th, 75th quartile). Correlations between maternal and PUFAs were assessed using Spearman's rank correlation coefficient.

Primary exposures, including fish intake (maternal and child), maternal individual PUFA, and cord blood individual PUFA concentrations, were grouped into quartiles for separate regression models. Logistic regression models estimated odds ratios (OR) and 95% confidence intervals (CI) for the association of each primary exposure separately with the dichotomous outcome, presence of asthma. Previous observational studies that investigated associations between diet, asthma, and allergy prevalence have used a range of covariates in their regression models. A systematic review of 62 observational studies proposed a set of covariates to be used in future statistical analysis.^28^ Accordingly, our logistic regression models were adjusted for seven potential confounders: maternal age, maternal BMI, gestational age, birth weight, Hollingshead socioeconomic status (SES), child sex, and child fish intake at 7 years. There were no mothers who reported smoking during pregnancy and few consumed alcoholic drinks (*n* = 3); therefore, these variables were not included in the regression models. Owing to the higher prevalence of asthma in male children compared to females observed in the literature,^4^ we then considered 2‐way interactions between maternal or cord PUFAs (in quartiles) and child sex (male/female) with asthma. Analyses using cord ALA and EPA concentrations were not completed as these PUFA values were below the lower limit of detection.

## 3 RESULTS

From 1535 mother–child pairs who were initially recruited, 923 mother–child dyads were included in final analyses (Figure 1). Maternal, neonatal, and child characteristics of the included participants are presented in Table 1, while descriptive characteristics for the original cohort are summarized in Table S1. Of the total number of children aged 7 years included in the final analysis, 97 (10.5%) met our criteria for having asthma.

**TABLE 1:** Maternal and child characteristic distribution in the included study population.

Variable	*n*	Mean ± SD	Minimum	Maximum
Mothers				
Maternal age at enrolment (y)	923	26.92 ± 6.41	16.03	46.56
Height (cm)	923	161.99 ± 5.91	143.00	180.00
Weight (kg)	923	71.52 ± 17.83	34.79	142.30
BMI (kg/m^2^)	923	27.27 ± 6.47	14.67	49.60
Estimated weekly fish meals	923	8.47 ± 4.55	0.00	37.00
SES	923	31.96 ± 10.56	11.00	63.00
Neonatal				
Gestational age (w)	923	39.03 ± 1.51	32.00	41.00
Birth weight (kg)	923	3.20 ± 0.49	1.56	5.20
Child				
Age (y)	923	7.42 ± 0.20	7.06	7.93
Weight (kg)	923	26.55 ± 6.70	13.00	64.60
Height (cm)	923	125.69 ± 5.86	91.00	142.50
Estimated weekly fish meals	923	6.00 ± 3.60	0.00	26.00
Sex (M/F)		483/440		
Asthmatic	97			
% Males with asthma		11.2%		
% Females with asthma		9.8%		

*Note*: Data represented as mean ± standard deviation, unless stated otherwise.

Abbreviations: BMI, body mass index; cm, centimeters; kg, kilogram; SES, Hollingshead socioeconomic status; w, weeks; y, years.

### 3.1 Fish consumption

The average fish consumption of mothers during pregnancy was 8 fish meals/week (Table 1); examples of fish commonly consumed by the Seychellois population are summarized in Table S2. No significant associations were observed between maternal fish intake (oily, lean, and total fish intake) and asthma prevalence at 7 years of age (Table 2). Child total fish consumption at 7 years was reported to be an average of 6 meals/week (Table 1). There were no significant associations observed between child oily fish and total fish consumption with asthma prevalence (Table 2). Consumption of 1–2 lean fish meals per week in childhood, compared to no lean fish consumption, was observed to be associated with reduced odds of asthma (OR 0.47, CI 0.24–0.95, *p* = .035) (Table 2). However, no significant associations were observed with lean fish consumption ≥3 meals per week compared to no lean fish meals.

**TABLE 2:** Fish consumption and its association with asthma prevalence at 7 years of age.

Fish intake		*n*	Unadjusted OR (95% CI)	*p*‐Value^a^	Adjusted OR^b^ (95% CI)	*p*‐Value^a^
*Maternal* (*total*/*asthmatic*)						
Oily fish	<2	184/22	Ref	–	Ref	–
	2–3	220/28	1.07 (0.59–1.95)	0.82	1.13 (0.61–2.06)	0.70
	4–5	222/21	0.77 (0.41–1.45)	0.42	0.78 (0.41–1.49)	0.45
	≥6	297/26	0.71 (0.39–1.29)	0.26	0.75 (0.41–1.39)	0.36
Lean fish	No fish	237/20	Ref	–	Ref	–
	1	135/10	0.87 (0.39–1.91)	0.73	0.85 (0.38–1.90)	0.70
	2–3	288/35	1.50 (0.84–2.68)	0.17	1.50 (0.84–2.70)	0.17
	≥4	263/32	1.50 (0.83–2.71)	0.18	1.70 (0.93–3.10)	0.08
Total fish	<5	159/14	Ref	–	Ref	–
	5–7	290/35	1.42 (0.74–2.73)	0.29	1.46 (0.75–2.83)	0.26
	8–10	232/22	1.10 (0.54–2.19)	0.82	1.12 (0.55–2.29)	0.75
	≥11	242/26	1.25 (0.71–2.47)	0.53	1.47 (0.73–2.96)	0.29
*Child*						
Oily fish	No fish	122/15	Ref	–	Ref	–
	1–2	327/33	0.80 (0.42–1.53)	0.50	0.81 (0.42–1.56)	0.53
	3	172/15	0.68 (0.32–1.45)	0.32	0.68 (0.32–1.45)	0.31
	≥4	302/34	0.91 (0.47–1.73)	0.76	0.98 (0.50–1.89)	0.94
Lean fish	No fish	524/63	Ref	–	Ref	–
	1–2	160/10	0.49 (0.24–0.98)	**0.042**	0.47 (0.24–0.95)	**0.035**
	≥3	239/24	0.82 (0.50–1.34)	0.43	0.84 (0.51–1.39)	0.50
Total fish	< 3	114/17	Ref	–	Ref	–
	3–4	266/27	0.65 (0.34–1.24)	0.19	0.62 (0.32–1.19)	0.15
	5–7	294/30	0.65 (0.34–1.23)	0.18	0.67 (0.35–1.28)	0.22
	≥8	249/23	0.58 (0.30–1.14)	0.11	0.63 (0.32–1.25)	0.18

*Note*: Bold values indicate statiscally significnat of *p* < 0.05.

^a^ Test for trend (Wald's chi‐square test).

^b^ Adjusted for maternal age, maternal BMI, Hollingshead socioeconomic status, gestation age, child sex, childbirth weight and child fish intake at 7 yr. (for maternal analysis), and maternal fish intake (for child fish intake analysis). Fish intake reported as meals per week. Total fish = sum of oily fish, lean fish, and other seafood products. *n* = 923.

### 3.2 Serum PUFA concentrations

No significant differences were observed in the median maternal PUFA status between asthmatic and non‐asthmatic mother–child dyads (Table 3). Maternal PUFA status concentrations were divided into quartiles and results from regression analyses found no significant associations with childhood asthma prevalence (Table 4). Maternal and cord blood DHA concentrations were positively in non‐asthmatic and asthmatic mother–child dyads (rho = 0.10, *p* = .004 and rho = 0.23, *p* = .027, respectively) (Table S3). The median concentration of DHA in cord blood was significantly higher in asthmatic children compared to non‐asthmatics (0.096 (0.074, 0.150) mg/mL vs. 0.088 (0.060, 0.121) mg/mL; *p* = .006) (Table 3). In cord blood, DHA concentrations in the highest quartile (≥0.123 mg/mL), compared to the lowest quartile (<0.061 mg/mL), were significantly associated with increased odds of childhood asthma (Table 5) (OR 2.21, CI 1.15–4.27, *p* = .018). Nevertheless, cord blood DHA concentrations in the other quartiles showed no significant associations with asthma (Table 5). No significant associations were observed for cord blood LA, AA, total n‐6, and n6: n3.

**TABLE 3:** Serum PUFA concentrations for non‐asthmatic and asthmatic mother–child dyads.

PUFA	Maternal			Cord blood		
	Non‐asthmatic	Asthmatic	*p*‐Value*	Non‐asthmatic	Asthmatic	*p*‐Value*
LA	0.888 (0.723, 1.054)	0.858 (0.718, 1.000)	0.23	0.151 (0.109, 0.199)	0.157 (0.115, 0.205)	0.30
AA	0.214 (0.171, 0.274)	0.210 (0.156, 0.260)	0.29	0.193 (0.145, 0.269)	0.202 (0.145, 0.269)	0.76
Total n‐6	1.110 (0.910, 1.312)	1.073 (0.915, 1.276)	0.22	0.362 (0.280, 0.498)	0.367 (0.281, 0.510)	0.78
ALA	0.035 (0.035, 0.039)	0.035 (0.035, 0.036)	0.35	n.d.	n.d.	
EPA	0.049 (0.049, 0.051)	0.049 (0.049, 0.053)	0.52	n.d.	n.d.	
DHA	0.195 (0.140, 0.255)	0.176 (0.138, 0.232)	0.13	0.088 (0.060, 0.121)	0.096 (0.074, 0.150)	**0.006**
Total n‐3	0.281 (0.226, 0.347)	0.262 (0.222, 0.320)	0.12	n/a	n/a	
n‐6: n‐3	3.889 (3.259, 4.570)	3.943 (3.470, 4.558)	0.50	3.339 (2.508, 6.655)	3.627 (2.631, 7.777)	0.23

*Note*: All results are in mg/mL and are given as median (25th, 75th). Bold values indicate statiscally significnat of *p* < 0.05.

Abbreviations: ALA, alpha‐linolenic acid; DHA, docosahexaenoic acid; EPA, eicosapentaenoic acid; LA, linoleic acid; n.d., not detectable.

* *p*‐Value obtained by Mann–Whitney *U*. Total n‐3 PUFA is defined as the sum of ALA + EPA + DHA; Total n‐6 PUFA is defined as the sum of LA + AA. *n* = 923.

**TABLE 4:** Maternal PUFA status during pregnancy and its association with asthma prevalence at 7 years of age.

PUFA		*n* (total/asthmatic)	Unadjusted OR (95% CI)	*p*‐Value^a^	Adjusted OR^b^ (95% CI)	*p*‐Value^a^
LA	<0.724	230/24	Ref	–	Ref	–
	0.724–0.884	232/29	1.23 (0.69–2.18)	0.49	1.22 (0.68–2.18)	0.51
	0.885–1.0496	231/24	0.99 (0.55–1.81)	0.99	0.99 (0.54–1.83)	0.99
	≥1.0497	230/20	0.82 (0.44–1.53)	0.53	0.82 (0.43–1.54)	0.53
AA	<0.169	231/27	Ref	–	Ref	–
	0.169–0.2137	229/24	0.89 (0.49–1.59)	0.68	0.96 (0.53–1.73)	0.89
	0.2138–0.2711	232/26	0.95 (0.54–1.69)	0.87	0.99 (0.55–1.77)	0.96
	≥0.2712	231/20	0.72 (0.39–1.32)	0.28	0.77 (0.41–1.43)	0.41
Total n‐6	<0.912	231/23	Ref	–	Ref	–
	0.912–1.102	231/34	1.56 (0.89–2.74)	0.12	1.54 (0.87–2.73)	0.14
	1.103–1.309	231/20	0.86 (0.46–1.61)	0.63	0.89 (0.47–1.68)	0.71
	≥1.310	230/20	0.86 (0.46–1.62)	0.64	0.87 (0.46–1.65)	0.67
ALA	<0.03492	209/22	Ref	–	Ref	–
	0.03492–0.03499	233/25	1.02 (0.56–1.87)	0.95	1.07 (0.58–1.97)	0.83
	0.0350–0.3807	250/32	1.25 (0.70–2.22)	0.45	1.19 (0.66–2.13)	0.56
	≥0.3808	231/27	0.72 (0.37–1.38)	0.32	0.71 (0.37–1.38)	0.32
EPA	<0.0488	125/13	Ref	–	Ref	–
	0.0488–0.0489	333/29	0.82 (0.41–1.64)	0.58	0.84 (0.42–1.68)	0.62
	0.0490–0.0515	234/28	1.17 (0.58–2.35)	0.66	1.21 (0.60–2.45)	0.60
	≥0.05156	231/27	1.14 (0.57–2.30)	0.71	1.22 (0.60–2.48)	0.59
DHA	<0.1402	231/25	Ref	–	Ref	–
	0.1402–0.1939	231/31	1.28 (0.73–2.24)	0.39	1.35 (0.76–2.38)	0.30
	0.1940–0.2501	230/27	1.10 (0.62–1.95)	0.76	1.16 (0.64–2.10)	0.63
	≥0.2502	231/14	0.53 (0.27–1.05)	0.07	0.59 (0.29–1.20)	0.14
Total n‐3	<0.2258	231/25	Ref	–	Ref	–
	0.2258–0.2805	229/30	1.24 (0.71–2.19)	0.45	1.29 (0.73–2.28)	0.38
	0.2806–0.3441	233/25	0.99 (0.55–1.78)	0.97	1.05 (0.58–1.91)	0.87
	≥0.3442	230/17	0.66 (0.35–1.25)	0.20	0.73 (0.38–1.42)	0.36
n6: n3	<3.2885	231/19	Ref	–	Ref	–
	3.2885–3.8949	230/25	1.36 (0.73–2.55)	0.34	1.29 (0.69–2.43)	0.43
	3.8950–4.5667	231/29	1.60 (0.87–2.95)	0.13	1.45 (0.78–2.69)	0.24
	≥4.5668	231/24	1.29 (0.69–2.43)	0.42	1.17 (0.61–2.22)	0.64

Abbreviations: AA, arachidonic acid; ALA, alpha‐linolenic acid; CI, confidence intervals; DHA, docosahexaenoic acid; EPA, eicosapentaenoic acid; LA, linoleic acid; OR, odds ratio.

^a^ Test for trend (Wald's chi‐square test).

^b^ Adjusted for maternal age, maternal BMI, maternal fish intake, Hollingshead socioeconomic status, gestation age, child sex, childbirth weight and child fish intake at 7 yr. Total n‐3 PUFA is defined as the sum of ALA + EPA + DHA; Total n‐6 PUFA is defined as the sum of LA + AA. PUFAs measured in mg/mL. *n* = 923.

**TABLE 5:** Cord blood PUFA concentrations and its association with asthma prevalence at 7 years of age.

PUFA		*n* (total/asthmatic)	Unadjusted OR (95% CI)	*p*‐Value^a^	Adjusted OR^b^ (95% CI)	*p*‐Value^a^
LA	<0.110	225/17	Ref	–	Ref	–
	0.110–0.1509	232/29	1.75 (0.932–3.28)	0.08	1.78 (0.94–3.35)	0.08
	0.151–0.198	233/25	1.47 (0.77–2.80)	0.24	1.49 (0.77–2.86)	0.24
	≥0.199	233/26	1.54 (0.81–2.92)	0.19	1.58 (0.82–3.01)	0.17
AA	<0.145	228/23	Ref	–	Ref	–
	0.145–0.193	233/23	0.98 (0.53–1.80)	0.94	0.98 (0.53–1.82)	0.96
	0.194–0.268	231/28	1.23 (0.69–2.21)	0.49	1.24 (0.69–2.24)	0.48
	≥0.269	231/23	0.99 (0.54–1.81)	0.99	0.99 (0.53–1.83)	0.97
Total n‐6	<0.280	226/20	Ref	–	Ref	–
	0.280–0.361	230/27	1.37 (0.74–2.52)	0.31	1.43 (0.77–2.65)	0.25
	0.362–0.499	236/24	1.17 (0.63–2.18)	0.63	1.22 (0.65–2.29)	0.54
	≥0.500	231/26	1.31 (0.71–2.41)	0.39	1.32 (0.71–2.45)	0.39
DHA	<0.061	222/15	Ref	–	Ref	–
	0.061–0.089	236/23	1.49 (0.76–2.94)	0.25	1.55 (0.78–3.09)	0.21
	0.090–0.122	231/28	1.90 (0.99–3.67)	0.06	1.94 (1.00–3.76)	0.05
	≥0.123	234/31	2.11 (1.11–4.02)	**0.024**	2.21 (1.15–4.27)	**0.018**
n6: n3	<2.526	230/22	Ref	–	Ref	–
	2.526–3.349	231/22	0.99 (0.54–1.85)	0.99	0.92 (0.49–1.74)	0.81
	3.350–6.868	230/26	1.21 (0.66–2.20)	0.54	1.21 (0.65–2.23)	0.55
	≥6.869	232/27	1.25 (0.69–2.26)	0.47	1.21 (0.67–2.22)	0.53

*Note*: Bold values indicate statiscally significnat of *p* < 0.05.

Abbreviations: AA, arachidonic acid; BMI, body mass index; CI, confidence intervals; DHA docosahexaenoic acid; LA linoleic acid; OR odds ratio.

^a^ Test for trend (Wald's chi‐square test).

^b^ Adjusted for maternal age, maternal BMI, maternal fish intake, Hollingshead socioeconomic status, gestation age, child sex, childbirth weight and child fish intake at 7 year. Total n‐6 PUFA is defined as the sum of LA + AA. PUFAs measured in mg/mL. *n* = 923.

### 3.3 Interactions

The associations between maternal and cord blood PUFA and asthma did not vary by child sex after adjusting for covariates (all interaction *p*‐values >0.05) (Tables S4 and S5).

## 4 DISCUSSION

In this high fish‐eating cohort, we identified 97 children (10.5%) who were self‐reported to be asthmatic. This finding is similar to the worldwide prevalence rate for childhood asthma, which is 11.6%, but lower than the prevalence found in some Western populations (12.62%).^3^, ^29^ Our data supports the growing body of evidence that consuming fish, even in high amounts, during pregnancy is not associated with asthma in children aged 7 years of age. In the current analysis, maternal PUFA status showed no significant associations with asthma prevalence. In cord blood, however, asthmatic children had significantly higher DHA concentrations compared to non‐asthmatic children, and concentrations of DHA ≥0.123 mg/mL (highest quartile) were significantly associated with increased risk of asthma prevalence at 7 years of age.

Some public health agencies recommend pregnant women to limit their fish intake due to the increase exposure of pollutants such as methylmercury and subsequent risk of adverse child development.^30^, ^31^ This may explain the few amounts consumed during pregnancy in previous birth‐cohort studies, such as 0.42 portions per week,^18^ and between 1 and 3 portions per month.^32^ The aforementioned studies also identified no significant associations with asthma prevalence in children aged up to 10 years of age. This is in agreement with our current findings, in which pregnant women consuming on average eight fish meals per week showed no significant association with childhood asthma prevalence. The Seychelles Ministry of Health recommend the population consume at least five fish meals per week,^33^ with no specific recommendations for pregnant women provided. Nonetheless, this high consumption of fish is unsurprising as the Seychellois population, including pregnant women rely on fish for their main source of protein.^23^ Recent evidence from the SCDS, however, has reported this cohort of pregnant women are consuming more foods associated with the “westernized diet”, such as chocolate and crisps, white fish, chicken and eggs.^34^ Further research should examine the dietary transition toward a “westernized” style in the Seychelles and possible influence on health conditions, such as asthma, increasing comparability to other populations worldwide.

Child total and oily fish consumption at 7 years were not significantly associated with asthma prevalence. Oily fish is high in n‐3 PUFAs, and a meta‐analysis of observational studies concluded intake of oily fish in early life was reported to have a prophylactic effect against current asthma in children aged 8–14 years.^35^ Lean fish, however, contains much lower quantities of n‐3 PUFAs compared to oily fish.^36^ Interestingly, our current analysis observed consumption of lean fish (1–2 meals per week) during childhood was significantly associated with reduced asthma prevalence when compared to no consumption of lean fish. Research is limited when investigating the consumption of lean fish with asthma prevalence; a Norwegian birth cohort identified no significant associations between lean fish and doctor‐diagnosed asthma prevalence at 2 years of age.^37^ Albeit authors observed consumption of 1 or more lean fish meals per week significantly reduced risk of eczema. This finding along with our own provides insight into the potential role of other components of fish (e.g., protein) and allergy development in childhood, warranting further investigation.

Fish is a rich source of n‐3 PUFA which are known to have anti‐inflammatory properties and are suggested to protect against the inflammatory processes involved in asthma.^6^ In our study of high fish consumers, we identified no association between maternal PUFA status and childhood asthma prevalence. These results are similar to those reported in the Netherlands from the Generation R (*n* = 4976) and in the KOALA (*n* = 1275) birth‐cohort studies which reported no significant associations between maternal PUFA status and asthma prevalence in children aged 6 years.^11^, ^12^ Maternal fish intake (meals/week) during pregnancy for these cohorts was reported to be 0.44 in Generation R and 0.98 in KOALA^38^ and lower compared to an average eight meals per week in the Seychelles cohort. This suggests a null effect of fish consumption on asthma development in the offspring regardless of low or high maternal fish consumption. However, a previous birth‐cohort study (*n* = 865) performed in the United Kingdom found that maternal n‐3 PUFA status, including EPA and DHA, was associated with reduced persistent wheeze in non‐atopic children aged 6 years^10^ and proposed intrauterine supply during fetal development is important. This study did not report maternal fish intake but noted that less than 1% of the cohort reported an intake of at least one fatty fish meal per week. It may be that the n‐3 PUFAs came from fish oil supplement use during pregnancy which has been shown to reduce the risk of offspring asthma.^19^, ^20^, ^21^

During fetal development, PUFAs are supplied to the fetus from the maternal stores and transferred through the umbilical cord as non‐esterified fatty acids derived from maternal triacylglycerols.^22^ In this current study, no significant associations were observed between cord blood n‐6 PUFA concentrations and asthma prevalence at 7 years of age. This finding is in agreement with several studies including the ALSPAC, Maastricht Essential Fatty Acid Birth (MEFAB), the Greek RHEA Mother–Child Study, and LISAplus birth cohorts which all reported no significant association between cord blood serum n‐6 PUFA and asthma prevalence in children 2–10 years of age.^39^, ^40^, ^41^ Similarly, in the FARMFLORA study, a Swedish birth cohort (*N* = 55) comparing diets and allergic predisposition of children raised on farms compared to non‐farmers, concluded cord blood AA concentrations were unrelated to allergic development.^42^

Recently published data from the FARMFLORA study reported that higher cord blood EPA concentration was associated with lower risk of asthma development at 3 and 8 years of age.^43^ The authors suggested that the protective effect of cord blood EPA is related to maternal fish intake during pregnancy. Fish intake during pregnancy was assessed using a food frequency questionnaire and reported to be 25 g per day for mothers of healthy children and 20 g per day for mothers of allergic children.^44^ The MEFAB and RHEA birth cohorts reported similar findings, in which n‐3 PUFAs (EPA + DHA) in cord blood were observed to be associated with reduced risk of asthma prevalence in children 6–7 years of age.^41^ In our analysis, the majority of EPA concentrations fell below the limit of detection in the cord samples from this cohort. This may be explained by the placental demand and uptake of DHA which is greater in the last trimester of pregnancy; therefore, maternal conversion of EPA to DHA is enhanced to meet the demands, thus less maternal EPA is available for placental transfer.^22^, ^45^

We observed higher concentrations of DHA in the cord blood of asthmatic children and cord DHA was associated with increased asthma prevalence at 7 years of age. Although this was unexpected, similar associations have been observed in a Swedish birth‐cohort study, in which higher concentrations of DHA in cord blood were identified in children with respiratory allergy, and total n‐3 PUFAs (consisting mostly of DHA) were associated with increased respiratory allergy development in children aged 13 years.^46^ The authors suggested that the enhanced transfer of PUFAs may be immune‐dampening and hamper the child's immune system development, resulting in allergy development. In our analysis, asthmatic mother–child dyads had a stronger correlation between maternal DHA and cord DHA than non‐asthmatic dyads. Our hypothesis is that the enhanced transfer of DHA may be in response to inflammation associated with allergic predisposition. This hypothesis is supported by previous, albeit limited, findings which identified associations between high DHA and reduced allergic immune response in cord blood mononuclear cells exposed to allergens.^47^

Epidemiological evidence has shown that asthma is more prevalent in males compared to females in childhood^4^ and sex‐differences in fatty acid metabolism exist through the lifespan.^48^ Nevertheless, research examining those differences in utero is less understood. In the CANDLE study, maternal n‐6 PUFA was identified to be strongly associated with asthma development in male children.^9^ Pro‐inflammatory prostaglandins and leukotrienes are derived from n‐6 PUFA and have been shown to be associated with asthma pathology.^6^ In this current analysis, no significant interactions were observed between child sex (male or female) and maternal or cord PUFA with childhood asthma.

This is the first study examining the associations between maternal fish intake, maternal PUFA status, and cord blood PUFA concentrations with asthma prevalence in childhood in a high fish‐eating population. A further strength is the use of the validated ISAAC questionnaire to assess asthma‐related outcomes which has previously been used in studies investigating associations between asthma prevalence and maternal diet during pregnancy. Furthermore, we determined PUFA concentrations in the form of triacylglycerols in serum and although difficult to compare to previous work, is representative of the transfer from mother to fetus via placenta. This cohort, however, was not recruited to investigate these associations as it was originally established to examine the associations between maternal fish consumption and child neurodevelopment.^24^ Moreover, 60% of the total eligible mother–child dyads were included in the final analysis. There were very little differences in mean averages for exposure variables between included participants and full cohort (Table 1 and Table S1, respectively), and we do not expect inclusion of full cohort would have influenced the observed associations. Nonetheless, a greater sample size, especially in terms of asthmatic children in a high fish‐eating population would strengthen the results. Clinical assessment/diagnosis of asthma in the children along with spirometry measures and biochemical analysis would greatly enhance these findings. Additionally, breastfeeding, parental history of asthma, and child PUFA status were not collected during recruitment and such information has previously been reported to influence childhood asthma.

## 5 CONCLUSION

This is the first report of childhood asthma prevalence in the Republic of Seychelles. In this report, we identified no significant associations between fish consumption or PUFA status during pregnancy and childhood asthma prevalence in a high fish‐eating population. Higher concentrations of DHA in cord serum were associated with increased asthma prevalence in the highest quartile of children 7 years of age, an unexpected result. Further work should explore this relationship of DHA and early life allergic sensitisation, particularly examining cord blood DHA concentrations in a larger cohort of asthmatic children. This study highlighted some beneficial associations between child fish intake and reduced asthma prevalence.

## AUTHOR CONTRIBUTIONS

**Cealan O. Henry:** Writing – original draft; visualization; methodology; investigation. **Philip J. Allsopp:** Supervision; writing – review and editing; conceptualization. **Alison J. Yeates:** Writing – review and editing; funding acquisition. **Toni Spence:** Writing – review and editing; investigation. **Marie C. Conway:** Writing – review and editing; investigation. **Maria S. Mulhern:** Writing – review and editing; funding acquisition. **Emelyn Shroff:** Writing – review and editing; resources; funding acquisition. **Conrad F. Shamlaye:** Funding acquisition; resources; writing – review and editing; conceptualization. **Juliette Henderson:** Resources; funding acquisition. **Edwin van Wijngaarden:** Funding acquisition; writing – review and editing; resources; conceptualization. **Gary J. Myers:** Funding acquisition; writing – review and editing; resources; conceptualization. **J. J. Strain:** Funding acquisition; writing – review and editing; conceptualization; resources. **Emeir M. McSorley:** Conceptualization; funding acquisition; writing – review and editing; supervision; resources.

## FUNDING INFORMATION

This study was supported by the National Institutes of Health (grants R01‐ES010219, P30‐ES01247, and T32‐ES007271) and in‐kind support from the government of Seychelles.

## CONFLICT OF INTEREST STATEMENT

The authors declare that there are no conflicts of interest.

## Supporting information


Table S1.
