# Supplementary material for: Associations between maternal fish intake and polyunsaturated fatty acid status with childhood asthma in a high fish‐eating population
Source: Pediatr Allergy Immunol. 2025 Jan 8;36(1):e70019. doi: 10.1111/pai.70019 (PMC11707734; doi:10.1111/pai.70019)
Supplement: Supplementary file 1 — Table S1. [file PAI-36-e70019-s001.docx]

**Supplementary table 1: Maternal and child characteristics of the original cohort**

| **Variable** | **n** | **Mean ± SD** | **Minimum** | **Maximum** |
| --- | --- | --- | --- | --- |
| Mothers  Maternal age at enrolment (y)  Height (cm)  Weight (kg)  BMI (kg/m^2^)  Estimated weekly fish meals  Total n-6 PUFA (mg/ml)  Total n-3 PUFA (mg/ml) | 1485  1487  1487  1487  1385  1448  1448 | 26.89 ± 6.29  161.91 ± 5.86  70.68 ± 17.19  26.93 ± 6.30  8.52 ± 4.51  1.10 ± 2.94  0.27 ± 0.09 | 16.03  143.00  34.79  14.67  0.00  0.43  0.12 | 46.56  181.00  142.30  49.65  37.00  2.71  0.64 |
| Neonatal  Gestational age (w)  Birth weight (kg)  Total n-6 PUFA (mg/ml)  DHA (mg/ml) | 1399  1409  1088  1088 | 39.06 ± 1.39  3.19 ± 0.46  0.45 ± 0.27  0.10 ± 0.05 | 34.00  1.62  0.10  0.01 | 41.00  5.20  2.46  0.50 |
| Child  Age (y)  Weight (kg)  Height (cm)  Estimated weekly fish meals  Sex (M/F) | 1451  1445  1445  1115 | 7.36 ± 0.21  26.17 ± 6.62  125.37 ± 5.76  5.96 ± 3.57  (788/718) | 7.00  13.00  91.00  0.00 | 7.90  64.60  142.50  26.00 |

Data represented as mean ± standard deviation, unless stated otherwise. Abbreviations: BMI, body mass index; cm, centimetres; kg, kilogram; mg, milligrams; ml, millilitres; w, weeks; y, years.

| **Fish species** | **Type** |
| --- | --- |
| Karang | Oily |
| Barracuda (bekin) | Lean |
| Fresh Tuna (ton) | Oily |
| Red snapper (bourzwa) | Lean |
| Grouper (vyey) | Lean |
| Parrot fish (kakatwa) | Lean |
| Spinefoot shoemaker (kordonnyen) | Lean |
| Mackerel (makro) | Oily |
| Emperor fish (baksou) | Lean |
| Job (zob) | Lean |
| Bonito (bonit) | Oily |
| Octopus (zourit) | Other seafood product |
| Tinned tuna (ton dan bwat) | Oily |
| Tinned sardines  (sardine dan bwat) | Oily |
| Shark (reken) | Lean |
| Salted fish (pwason sale) | Lean |
| Prawns (kanmaron) | Other seafood product |
| Crab/lobster (krab/oumar) | Other seafood product |

**Supplementary table 2: Examples of fish species commonly consumed in the cohort**

**Supplementary table 3: Correlation of maternal and cord blood PUFA concentrations**

| **PUFA** | **Non-Asthmatic** | | **Asthmatic** | |
| --- | --- | --- | --- | --- |
|  | rho* | P-value | rho* | p-value |
| LA | 0.05 | 0.15 | 0.24 | **0.017** |
| AA | 0.03 | 0.38 | 0.08 | 0.41 |
| Total n-6 | -0.04 | 0.31 | 0.22 | **0.033** |
| DHA | 0.10 | **0.004** | 0.23 | **0.027** |
| n6: n3 | 0.03 | 0.38 | 0.19 | 0.06 |

*Spearman correlation coefficients between maternal status and cord blood concentrations. Total n6 – sum of LA and AA. Abbreviations: LA, linoleic acid; AA, arachidonic acid; DHA, docosahexaenoic acid. PUFA measured in mg/ml.

**Supplementary table 4: The effect of child sex on the associations between maternal PUFA status and childhood asthma prevalence**

| **PUFA** | | **Male (n=483)** | | | **Female (n=440)** | | |
| --- | --- | --- | --- | --- | --- | --- | --- |
|  |  | **p-value for interaction** | **OR (95% CI)** | **p-value*** | **p-value for interaction** | **OR (95% CI)** | **p-value*** |
| LA | < 0.724  0.724-0.884  0.885-1.0496  ≥1.0497 | 0.29 | Ref  1.69 (0.96-2.95)  1.06 (0.54-2.06)  0.92 (0.45-1.90) | 0.07  0.87  0.83 | 0.746 | Ref  0.77 (0.37-1.60)  0.94 (0.48-1.80)  0.72 (0.35-1.45) | -  0.48  0.84  0.35 |
| AA | < 0.169  0.169-0.2137  0.2138-0.2711  ≥0.2712 | 0.05 | Ref  1.23 (0.63-2.40)  2.11 (1.21-3.69)  0.90 (0.44-1.83) | -  0.55  0.01  0.76 | 0.108 | Ref  0.85 (0.44-1.64)  0.28 (0.10-0.79)  0.78 (0.38-1.57) | -  0.63  0.02  0.49 |
| Total n-6 | < 0.912  0.912-1.102  1.103-1.309  ≥1.310 | 0.05 | Ref  2.03 (1.17-3.50)  1.09 (0.56-2.13)  0.83 (0.39-1.75) | -  0.01  0.79  0.62 | 0.640 | Ref  0.97 (0.50-1.86)  0.62 (0.29-1.34)  0.81 (0.41-1.60) | -  0.92  0.23  0.54 |
| ALA | < 0.03492  0.03492-0.03499  0.0350-0.3807  ≥0.3808 | 0.18 | Ref  1.41 (0.80-2.50)  1.00 (0.53-1.87)  0.50 (0.22-1.14) | -  0.25  0.99  0.10 | 0.452 | Ref  0.67 (0.30-1.52)  1.40 (0.79-2.48)  0.97 (0.49-1.93) | -  0.34  0.25  0.94 |
| EPA | < 0.0488  0.0488-0.0489  0.0490-0.0515  ≥0.05156 | 0.49 | Ref  0.99 (0.55-1.81)  1.50 (0.81-2.78)  1.38 (0.74-2.55) | -  0.98  0.20  0.31 | 0.616 | Ref  0.65 (0.35-1.23)  0.90 (0.46-1.74)  1.00 (0.50-1.99) | -  0.19  0.75  1.00 |
| DHA | < 0.1402 | 0.20 | Ref | - | 0.299 | Ref | - |

**Supplementary table 4: continued**

| **PUFA** | | **Male (n=483)** | | | **Female (n=440)** | | |
| --- | --- | --- | --- | --- | --- | --- | --- |
|  |  | **p-value for interaction** | **OR (95% CI)** | **p-value*** | **p-value for interaction** | **OR (95% CI)** | **p-value*** |
|  | 0.1402-0.1939  0.1940-0.2501  ≥0.2502 |  | 1.43 (0.79-2.56)  1.54 (0.85-2.78)  0.68 (0.31-1.50) | 0.24  0.16  0.34 |  | 1.26 (0.67-2.35)  0.80 (0.39-1.61)  0.50 (0.21-1.18) | 0.48  0.53  0.11 |
| Total n-3 | < 0.2258  0.2258-0.2805  0.2806-0.3441  ≥0.3442 | 0.516 | Ref  1.35 (0.74-2.45)  1.42 (0.77-2.61)  0.89 (0.43-1.83) | -  0.33  0.26  0.76 | 0.363 | Ref  1.22 (0.65-2.28)  0.68 (0.34-1.47)  0.58 (0.26-1.30) | -  0.53  0.35  0.19 |
| n6: n3 | < 3.2885  3.2885-3.8949  3.8950-4.5667  ≥4.5668 | 0.474 | Ref  1.60 (0.88-2.89)  1.24 (0.67-2.31)  1.08 (0.55-2.11) | -  0.12  0.50  0.82 | 0.601 | Ref  0.66 (0.31-1.43)  1.22 (0.66-2.29)  0.89 (0.46-1.74) | -  0.29  0.53  0.74 |

*Test for trend (Wald’s chi-square test). Total n-3 PUFA is defined as the sum of ALA + EPA + DHA; Total n-6 PUFA is defined as the sum of linoleic acid + AA. Models shown were adjusted for maternal age, maternal BMI, maternal fish intake, Hollingshead socioeconomic status, gestation age, childbirth weight and child fish intake. Abbreviations: AA, arachidonic acid; ALA, alpha-linolenic acid; DHA, docosahexaenoic acid; EPA eicosapentaenoic acid; OR odds ratio. PUFAs measured in mg/ml. n=923.

**Supplementary table 5: The effect of child sex on the associations between cord blood PUFA status and childhood asthma prevalence**

| **PUFA** | | **Male (n=483)** | | | **Female (n=440)** | | |
| --- | --- | --- | --- | --- | --- | --- | --- |
|  |  | **p-value for interaction** | **OR (95% CI)** | **p-value*** | **p-value for interaction** | **OR (95% CI)** | **p-value*** |
| LA | < 0.110  0.110-0.1509  0.151-0.198  ≥0.199 | 0.198 | Ref  1.73 (0.95-3.15)  1.27 (0.65-2.50)  1.62 (0.90-2.93) | -  0.07  0.49  0.11 | 0.905 | Ref  0.96 (0.50-1.86)  0.93 (0.49-1.76)  0.75 (0.36-1.59) | -  0.90  0.82  0.46 |
| AA | < 0.145  0.145-0.193  0.194-0.268  ≥0.269 | 0.154 | Ref  1.31 (0.69-2.49)  1.90 (1.07-3.36)  0.98 (0.49-1.95) | -  0.42  0.03  0.96 | 0.517 | Ref  0.66 (0.32-1.36)  0.65 (0.31-1.36)  0.92 (0.48-1.78) | -  0.26  0.26  0.80 |
| Total n-6 | < 0.280  0.280-0.361  0.362-0.499  ≥0.500 | 0.391 | Ref  1.67 (0.92-3.04)  1.24 (0.65-2.37)  1.26 (0.67-2.38) | -  0.09  0.51  0.47 | 0.918 | Ref  0.82 (0.40-1.66)  0.83 (0.42-1.65)  0.97 (0.50-1.89) | -  0.58  0.60  0.94 |
| DHA | < 0.061  0.061-0.089  0.090-0.122  ≥0.123 | 0.153 | Ref  1.18 (0.57-2.38)  1.44 (0.80-2.61)  1.92 (1.07-3.43) | -  0.64  0.23  0.03 | 0.947 | Ref  0.88 (0.46-1.69)  1.14 (0.57-2.28)  1.04 (0.54-2.03) | -  0.70  0.71  0.90 |
| n6: n3 | < 2.526  2.526-3.349  3.350-6.868  ≥6.869 | 0.334 | Ref  1.16 (0.62-2.20)  1.78 (0.96-3.31)  1.20 (0.64-2.26) | -  0.64  0.07  0.57 | 0.421 | Ref  0.61 (0.28-1.32)  0.69 (0.34-1.39)  1.11 (0.58-2.13) | -  0.21  0.29  0.75 |

*Test for trend (Wald’s chi-square test). Total n-6 PUFA is defined as the sum of LA + AA. Models shown were adjusted for maternal age, maternal BMI, maternal fish intake, Hollingshead socioeconomic status, gestation age, childbirth weight and child fish intake. Abbreviations: AA, arachidonic acid; ALA, alpha-linolenic acid; DHA docosahexaenoic acid; LA, linoleic acid; OR odds ratio. PUFAs measured in mg/ml. n=923.
